# AI/ML-Based Medical Image Processing and Analysis

**DOI:** 10.3390/diagnostics13243671

**Published:** 2023-12-14

**Authors:** Jaafar Alghazo, Ghazanfar Latif

**Affiliations:** 1 Artificial Intelligence Research Initiative, College of Engineering and Mines, University of North Dakota, Grand Forks, ND 58202, USA; jaafar.alghazo@und.edu; 2 Computer Science Department, Prince Mohammad Bin Fahd University, Khobar 34754, Saudi Arabia

The medical field is experiencing remarkable advancements, notably with the latest technologies—artificial intelligence (AI), big data, high-performance computing (HPC), and high-throughput computing (HTC)—that are in place to offer groundbreaking solutions to support medical professionals in the diagnostic process. Among these innovations, the field of radiology is receiving the most attention from researchers, where a significant number of AI/machine learning (ML) medical software and devices have already gained approval from the U.S. Food and Drug Administration (FDA) [1]. While many AI-driven solutions primarily focus on radiology, their potential reaches across various facets of the medical field. This also opens the doors for researchers to study the use of these technologies in other categories in the medical field.

Early diagnosis is essential in medicine, as it has repeatedly demonstrated its ability to save lives and extend patient lifespans [2,3,4]. However, traditional visual diagnosis is often a time-consuming and costly process, demanding the use of specialized equipment and the expertise of skilled medical professionals. In contrast, automatic diagnosis, although still in its early stages, promises to be more cost-effective and efficient, increasing the accuracy of the diagnosis process. Advancements in the area of automatic diagnosis using integrated, cutting-edge technologies can significantly increase the number of patients screened for potential diagnoses. It is worth noting that, while automatic diagnosis leverages AI and other technologies, it still requires the validation and oversight of medical experts to ensure its accuracy and safety [5,6,7].

There is a surge of research efforts integrating AI, ML, and deep learning (DL) into the diagnosis process. This Special Issue sought to inspire researchers to take this integration a step further by incorporating newer technologies, such as the Internet of Things (IoT), cloud computing, big data, HTC, HPC, etc., into the medical diagnosis landscape. These interdisciplinary research endeavors hold the potential to lay the foundation for innovative devices and systems that could ultimately receive patents and approval for use in the medical field.

The collaborative exploration of AI, ML, DL, HCT, HPC, IoT, cloud computing, big data, and other cutting-edge technologies in medical diagnosis is a testament to the dynamic nature of healthcare innovation. It highlights the potential for transformative breakthroughs in diagnosing and treating medical conditions, ultimately benefiting patients and healthcare professionals alike. Through the tremendous efforts of the research community, the AI/ML-based medical image processing and analysis issue received outstanding submissions that add to the current body of knowledge.

In contribution 1, the authors presented an innovative technique for rapid identification and classification of histopathology images of lung tissue using convolutional neural networks (CNNs) with fewer parameters, optimized by the light gradient boosting model (LightGBM) classifier. They reported an accuracy of 99.6% when testing on the LC25000 dataset. In contribution 2, the authors propose a deep-learning solution to classify four lung diseases using X-ray images. Their method is based on the EfficientNet B7 model, followed by fine-tuned layers and hyperparameters. They reported an average test accuracy of 97.42%, a sensitivity of 95.93%, and a specificity of 99.05%. In contribution 3, the authors propose a method for isolating and detecting brain tumors using noisy MRI brain images, anisotropic noise removal filtering, segmentation with an SVM classifier, and isolation of the adjacent region from the normal morphological processes. They reported that the SVM could partition data with 98% accuracy.

In contribution 4, the authors propose a radiomics model for discriminating early from later stages of nasopharyngeal carcinoma (NPC) tumors, which are common in China. They reported an area under the curve (AUC) of 0.847, which they indicated produced better results than visual assessments. In contribution 5, the authors propose using deep learning to automatically segment the parotid gland on computer tomography images. They reported an area under the curve (AUC) of 0.96 and proved that the parotid gland can be segmented using the deep learning method. In contribution 6, the authors investigate the effects of combinations of different preprocessing algorithms on the detection of breast cancer. They reported that, by comparing the performances of the classification methods, different preprocessing algorithms effectively detected the presence of breast lesions and distinguished benign from malignant ones. In contribution 7, the authors propose an end-to-end CNN transformer hybrid model with a focal loss (FL) function for classifying skin lesion images. They tested this using the 2018 international skin imaging collaboration (ISIC) dataset and reported an accuracy of 89.48%.

In contribution 8, the authors propose a novel, anatomy-sensitive retinal vessel segmentation framework to preserve instance connectivity and improve the segmentation accuracy of thin retinal vessels. TransUNet is the backbone of their framework. They tested their proposed framework on three public datasets: DRIVE, CHASE-DB1, and STARE. They reported an improvement of the F1 scores by 0.36% and 0.31%, respectively, for the DRIVE and CHASE-DB1 datasets. In contribution 9, the authors propose a method for detecting the stages of diabetic retinopathy. Their approach is a hybrid based on image preprocessing and ensemble features. They reported that the support vector machine (SVM) classifier achieved the highest classification accuracy of 98.85% on a publicly available dataset, i.e., Kaggle EyePACS. In contribution 10, the authors a modified a convolutional neural network (CNN) for diabetic retinopathy detection using fundus images at the Sindh Institute of Ophthalmology and Visual Sciences. They reported 93.72% accuracy, 97.30% sensitivity, and 92.90% specificity when tested on a small private dataset with data for 398 patients.

In contribution 11, the authors propose a new approach, CAD-ALZ, for the recognition of multistage Alzheimer’s in which deep features were extracted through the ConvMixer layer with a block-wise fine-tuning method on a small original dataset. They reported a sensitivity of 99.69% and an F1-score of 99.61% for this method. In contribution 12, the authors propose a method of Alzheimer’s disease classification using transfer learning. They reported an overall accuracy of 97.84%.

In contribution 13, the authors applied six pre-trained DNN models, namely, VGG16, VGG19, ResNet101, MobileNetV2, InceptionResNetV2, and DenseNet121 for knee osteoarthritis (KOA) diagnosis using images obtained from the Osteoarthritis Initiative (OAI) dataset. They achieved maximum classification accuracies of 69%, 83%, and 89%, respectively, with the ResNet101 DNN model. In contribution 14, the authors evaluated the deep learning performance of 18F-FDG PET-CT images to predict overall survival in HCC patients before liver transplantation (LT). Their proposed tool could be a predictive tool that can effectively determine prognosis and select optimal candidates for transplants. In contribution 15, the authors propose a diagnostic method for cardiovascular disorders (CVDs) based on phonocardiogram (PCG) signals. They offer a novel patch-embedding technique (CVD-Trans) based on convolutional vision transformers. They reported an accuracy of 99%. In contribution 16, the author proposes using log transformation and power law transformation to achieve contract and illumination for the purpose of detecting gastrointestinal (GI) diseases. Testing on the KVASIR dataset achieved an accuracy of 96.71%.

This comprehensive compilation of research articles targeting automatic diagnosis and other relevant medically related areas where AI, machine learning, and deep learning are contributing to supporting medical experts is an excellent resource and an excellent milestone towards finding optimal solutions to be practically used in hospitals and clinics. It was noticed that the contributions ranged from lung cancer, brain cancer, and breast cancer diagnosis to pneumonia, pneumothorax, and tuberculosis. Other research concentrated on Alzheimer’s, diabetic retinopathy, and knee osteoarthritis. It should be noted that these are but a few articles that have extensively researched these areas and the use of AI in the medical field.

Research in AI and medicine is moving toward the achievement of an AI doctor who can initially diagnose some minor medical conditions [8,9,10]. It should be noted that the research community almost agrees that medical diagnosis can never rely solely on AI, as there always needs to be a human in the loop in the form of a medical expert. This applies to the use of AI in medicine or in any other field [11,12,13]. Therefore, we urge the research community to follow this approach until a time comes when humans can trust AI to automatically make life-threatening decisions without human intervention.

We thank the contributors to this special edition, and there will be future special editions that are of interest to the research community and add to the body of knowledge.

## References

[1] Center Artificial Intelligence and Machine Learning (AI/ML)—Enabled Medical Devices. https://www.fda.gov/medical-devices/software-medical-device-samd/artificial-intelligence-and-machine-learning-aiml-enabled-medical-devices.

[2] García-Pola M., Pons-Fuster E., Suárez-Fernández C., Seoane-Romero J.M., Romero-Méndez A., López-Jornet P. (2021). Role of Artificial Intelligence in the Early Diagnosis of Oral Cancer. A Scoping Review. Cancers.

[3] Khodabandehloo E., Riboni D., Alimohammadi A. (2021). HealthXAI: Collaborative and Explainable AI for Supporting Early Diagnosis of Cognitive Decline. Future Gener. Comput. Syst..

[4] Latif G., Iskandar D.A., Alghazo J., Butt M.M. (2021). Brain MR Image Classification for Glioma Tumor Detection Using Deep Convolutional Neural Network Features. Curr. Med. Imaging Rev..

[5] Fukuda H., Ishihara R., Kato Y., Matsunaga T., Nishida T., Yamada T., Ogiyama H., Horie M., Kinoshita K., Tada T. (2020). Comparison of Performances of Artificial Intelligence versus Expert Endoscopists for Real-Time Assisted Diagnosis of Esophageal Squamous Cell Carcinoma (with Video). Gastrointest. Endosc..

[6] Chen J., Dhaliwal G., Yang D.S. (2022). Decoding Artificial Intelligence to Achieve Diagnostic Excellence. JAMA.

[7] Patel B.N., Rosenberg L., Willcox G., Baltaxe D., Lyons M., Irvin J., Rajpurkar P., Amrhein T.J., Gupta R.T., Halabi S. (2019). Human–Machine Partnership with Artificial Intelligence for Chest Radiograph Diagnosis. npj Digit. Med..

[8] Das S.R., Biswas S., Paul A., Dey A. (2017). AI Doctor: An Intelligent Approach for Medical Diagnosis. Lect. Notes Netw. Syst..

[9] Wang D., Wang L., Zhang Z., Wang D., Zhu H., Gao Y.Y., Fan X., Tian F. “Brilliant AI Doctor” in Rural Clinics: Challenges in AI-Powered Clinical Decision Support System Deployment. Proceedings of the 2021 CHI Conference on Human Factors in Computing Systems.

[10] Latif G., Shankar A., Alghazo J.M., Kalyanasundaram V., Boopathi C.S., Jaffar M.A. (2019). I-CARES: Advancing Health Diagnosis and Medication through IoT. Wirel. Netw..

[11] Budd S., Robinson E.C., Kainz B. (2021). A Survey on Active Learning and Human-In-The-Loop Deep Learning for Medical Image Analysis. Med. Image Anal..

[12] Fosch-Villaronga E., Khanna P., Drukarch H., Custers B. (2021). A Human in the Loop in Surgery Automation. Nat. Mach. Intell..

[13] Cardia L., Sierra-Franco C.A., Raposo A. Enabling Autonomous Medical Image Data Annotation: A Human-In-The-Loop Reinforcement Learning Approach. Proceedings of the 16th Conference on Computer Science and Intelligence Systems.

